# nucleAIzer: A Parameter-free Deep Learning Framework for Nucleus Segmentation Using Image Style Transfer

**DOI:** 10.1016/j.cels.2020.04.003

**Published:** 2020-05-07

**Authors:** Reka Hollandi, Abel Szkalisity, Timea Toth, Ervin Tasnadi, Csaba Molnar, Botond Mathe, Istvan Grexa, Jozsef Molnar, Arpad Balind, Mate Gorbe, Maria Kovacs, Ede Migh, Allen Goodman, Tamas Balassa, Krisztian Koos, Wenyu Wang, Juan Carlos Caicedo, Norbert Bara, Ferenc Kovacs, Lassi Paavolainen, Tivadar Danka, Andras Kriston, Anne Elizabeth Carpenter, Kevin Smith, Peter Horvath

**Affiliations:** 1 Synthetic and Systems Biology Unit, Hungarian Academy of Sciences, Biological Research Center (BRC), Temesvári körút 62, Szeged 6726, Hungary; 2 Doctoral School of Biology, University of Szeged, Közép fasor 52, Szeged 6726, Hungary; 3 Doctoral School of Computer Science, University of Szeged,Árpád tér 2, Szeged 6720, Hungary; 4 Doctoral School of Interdisciplinary Medicine, University of Szeged, Koranyi fasor 10, Szeged 6720, Hungary; 5 Doctoral School of Informatics, Eötvös Loránd University, Pázmány Péter sétány 1/C, Room 2.317, Budapest 1117, Hungary; 6 Imaging Platform, Broad Institute of Harvard and MIT, 415 Main Street, Cambridge, MA 02142, USA; 7 Institute for Molecular Medicine Finland (FIMM), University of Helsinki, Tukholmankatu 8, Helsinki 00014, Finland; 8 Single-Cell Technologies Ltd, Szeged 6726, Hungary; 9 KTH Royal Institute of Technology, School of Computer Science and Communication, Lindstedtsvägen 3, Stockholm 10044, Sweden; 10 Science for Life Laboratory, Solna, Sweden; 11 Lead Contact

## Abstract

Single-cell segmentation is typically a crucial task of image-based cellular analysis. We present nucleAIzer, a deep-learning approach aiming toward a truly general method for localizing 2D cell nuclei across a diverse range of assays and light microscopy modalities. We outperform the 739 methods submitted to the 2018 Data Science Bowl on images representing a variety of realistic conditions, some of which were not represented in the training data. The key to our approach is that during training nucleAIzer automatically adapts its nucleus-style model to unseen and unlabeled data using image style transfer to automatically generate augmented training samples. This allows the model to recognize nuclei in new and different experiments efficiently without requiring expert annotations, making deep learning for nucleus segmentation fairly simple and labor free for most biological light microscopy experiments. It can also be used online, integrated into CellProfiler and freely downloaded at www.nucleaizer.org.

A record of this paper’s transparent peer review process is included in the Supplemental Information.

## In Brief

Microscopy image analysis of single cells can be challenging but also eased and improved. We developed a deep learning method to segment cell nuclei. Our strategy is adapting to unexpected circumstances automatically by synthesizing artificial microscopy images in such a domain as training samples.

## Graphical Abstract

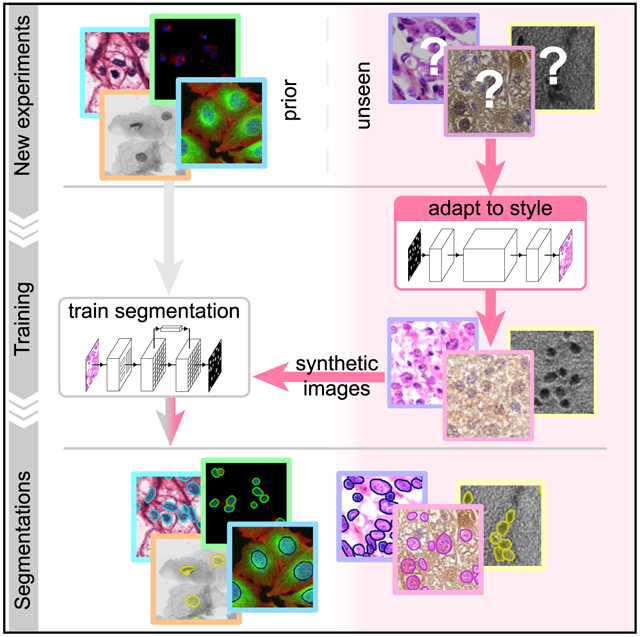

## INTRODUCTION

Identifying nuclei is the starting point for many microscopy-based cellular analyses, which are widespread in biomedical research. Accurate localization of the nucleus is the basis of a variety of quantitative measurements of important cell functions but is also a first step for identifying individual cell borders, which enables a multitude of further analyses. Until recently, the dominant approaches for this task have been based on classic image processing algorithms (e.g., thresholding and seeded water-shed; Carpenter et al., 2006), guided by shape and spatial priors (Molnar et al., 2016). These methods require expert knowledge to properly adjust the parameters, which typically must be retuned when experimental conditions change.

Recently, deep learning has revolutionized an assortment of tasks in image analysis, from image classification (Krizhevsky et al., 2017) to face recognition (Taigman et al., 2014) and scene segmentation (Badrinarayanan et al., 2017). It is also responsible for breakthroughs in diagnosing retinal images (De Fauw et al., 2018), classifying skin lesions with superhuman performance (Esteva et al., 2017), and correcting artifacts in fluorescence images (Weigert et al., 2017). Initial work (reviewed in Moen et al., 2019) indicates that deep learning is effective for nucleus segmentation (Falk et al., 2019; Van Valen et al., 2016; Cui et al., 2018); however, these methods often fail to properly separate touching nuclei well and most importantly lack robustness to unseen domains.

The 2018 Data Science Bowl (DSB) organized by Kaggle, Booz Allen Hamilton, and the Broad Institute challenged participants to push the state of the art in nucleus segmentation. The goal of the challenge was to develop fully automated and robust methods effective in a variety of conditions, including differing cell lines, treatments, and types of light microscopy. The challenge attracted thousands of data scientists from around the world. Approaches using deep learning dominated the competition, achieving scores that shattered what was previously possible: the best performing traditional methods we submitted ranked no higher than 1,000 out of 3,891 submissions in stage 1 (data not shown); even classical methods hand-tuned to five subsets of the testing data were beaten by 85 out of 739 submissions in stage 2 testing (Caicedo et al., 2019b). The top deep-learning-based methods relied on only a handful of different architectures, namely Mask R-CNN, U-Net, and feature-pyramid networks; the factors that participants commonly believed had most influence over their method’s ranking were the amount of data, the pre-processing, and methods used to augment the data.

**Figure 1.**
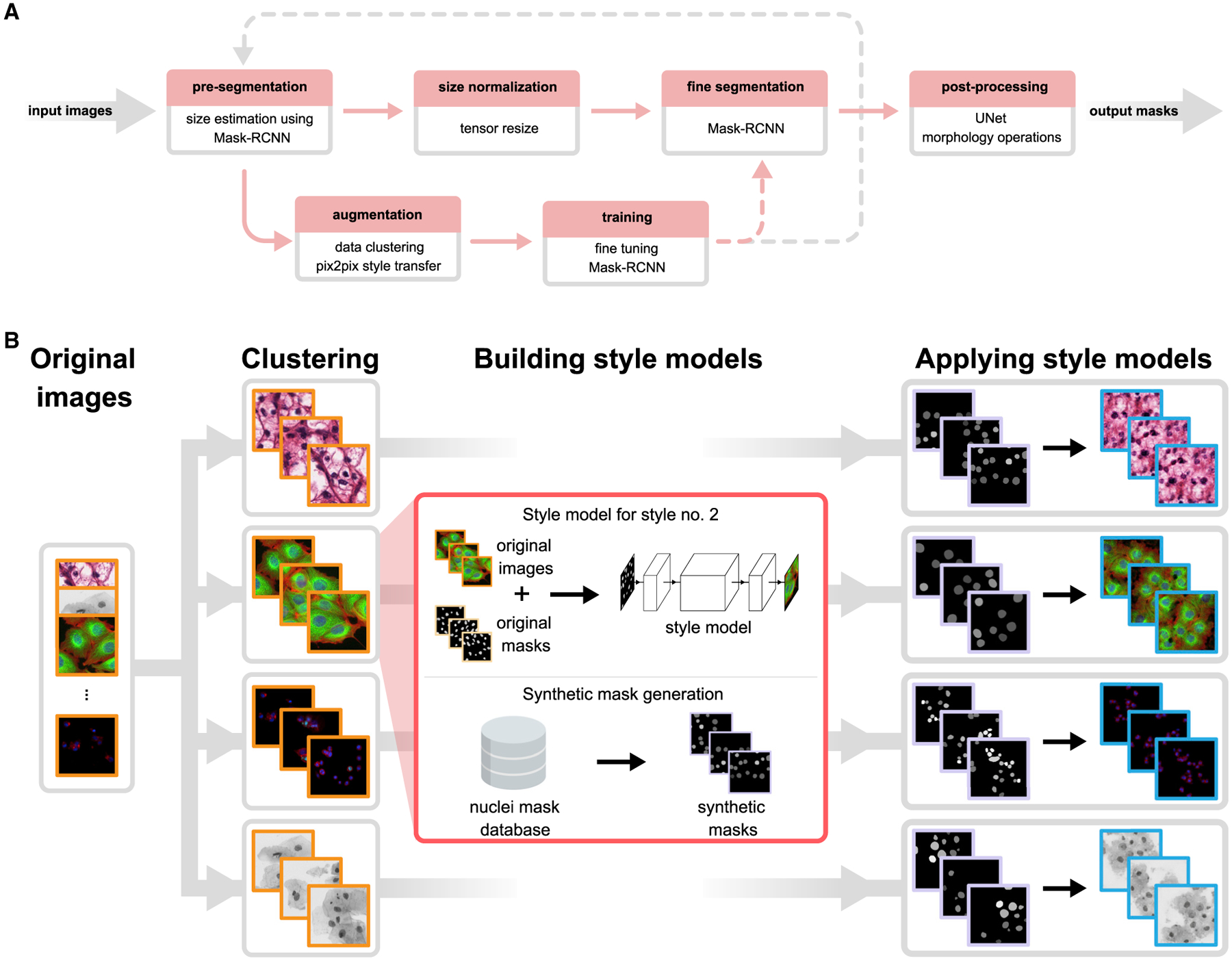
Overview of Our Approach (A) Upper row of boxes presents the nucleus segmentation and pre-processing; an initial Mask R-CNN network estimates typical nucleus sizes, then images are rescaled such that mean nucleus size is uniform and a Mask R-CNN network trained on images with uniform nucleus size predicts segmentations. A contour refinement step using a U-Net-based network with a morphology operation is applied to obtain the final segmentation result. The data augmentation pipeline is depicted in the bottom row, the training set is augmented with an artificially generated set of image/label pairs in the target domain(s), and a pre-trained Mask R-CNN method is fine-tuned using the augmented images. Augmentation and training steps may be iteratively repeated as the gray dashed line suggests. Upper row depicts the inference pipeline; bottom row, training. Solid lines indicate data flow; dashed lines indicate transfer of a trained model. (B) Image style-transfer-based data augmentation. To adapt our model to handle out-of-domain image types for which we have no segmentation labels, we synthesize new training data by first clustering images into similar groups, then learn a style transfer model. The style transfer model is provided with simulated nucleus masks, which mimic the number, shape, and size of the unseen nuclei, and then synthetic training image/label pairs are generated using the masks and the style transfer models. These data are added to the standard training data provided to Mask R-CNN, and the network learns to segment nuclei in the new domain. See also Figure S1.

We present here a superior approach we named nucleAIzer, which, unlike the previous best submissions, applies image style transfer (Isola et al., 2017): an image-to-image translation using a pixel-wise mapping from one image to the other that ensures the generated synthetic output image resembles the original as closely as possible. It aims to overcome one of the greatest challenges of deep learning, the extent of the annotated training set. In particular, we address the unsupervised domain adaptation problem in which the target (test) samples are drawn from a different distribution than the labeled training samples, but we have access to some unlabeled samples from the target distribution. We augment the training samples by creating realistic-looking artificial sample images with the texture, coloration, and pattern elements from source images not included in the training set using image style transfer (Figure 1). Combining this with a segmentation network based on Mask R-CNN (He et al., 2017), an instance segmentation and classification network, along with boundary correction using U-Net (Ronneberger et al., 2015), a semantic segmentation network for biomedical images, (Figure S4) and mathematical morphology, our method outperforms all other methods reported on the final DSB leaderboard (post-competition) (Our method achieved the top-score after the competition ended. An early version of our approach placed 27th out of 739 submissions in round 2 of the competition). We also demonstrate that our method outperforms similar baselines on public fluorescent and histology datasets. Our trained model does not require parameter tuning or specialized knowledge to use and can be applied on a wide variety of conditions and imaging modalities.

Our software is open source and freely available (Data S1 at https://github.com/spreka/biomagdsb). Pre-trained networks for DSB, fluorescent, and histology data can be applied to new images via CellProfiler (Data S2 and at https://github.com/CellProfiler/CellProfiler-plugins/blob/master/nucleaizer.py) or through an online interface at www.nucleaizer.org.

Our approach (Figures 1A and S1; STAR Methods) begins by automatically rescaling the images such that nucleus size is approximately uniform, as the performance of the network is improved if the nucleus size is fixed during training and inference (see STAR Methods; Figures S3 and S6). To do this, we estimate the typical nucleus size in the provided images with a Mask R-CNN-based network pretrained on a large set of diverse images with nucleus segmentations and fine-tuned using the provided training data and label masks. The output of this network is an initial segmentation we use to estimate the typical nucleus size. Alternatively, if the typical nucleus size is known a priori, it can be provided manually and the images rescaled accordingly.

Next, to adapt our model to handle a wide variety of cell types, staining methods, and imaging modalities, even those for which no segmentation annotations are available, we augment the training set with an artificially generated set of representative image-label pairs. This is accomplished using image style transfer. Training and inference both begin by automatically clustering training images into similar styles based on their appearance, using *k*-means (see STAR Methods; Figure 1B). For each cluster of similar image types, a style transfer network (Isola et al., 2017) is trained to generate synthetic images of the desired style with nuclei at specified locations. During training, nucleus annotations are used to train the style transfer network; during inference on out-of-domain target images, we use nucleus masks output from the initial segmentation network. After a style transfer network is trained for each image style, we generate a set of artificial nucleus masks representative of the shape, size, and spatial distribution of nuclei belonging to that style. For this, we used ~100,000 manually labeled single nucleus masks from the DSB set. A subset of these nuclei is selected that represent the shape distribution of the original morphologies, and they are placed such that they follow the spatial distribution of the image style (see STAR Methods). With trained style networks and representative nucleus masks in hand, we generate synthetic images in the desired style nearly indistinguishable from real microscopy images (see STAR Methods) with nuclei in locations defined by the artificial masks. The synthetic image-mask pairs make up the augmented dataset; samples are shown in Figures 1B and S7A. The augmented data are added to the training data for the segmentation network and further extended with conventional augmentations (rotation, cropping, intensity stretching, etc., see STAR Methods). For this experiment, we generated 20 synthetic image/mask pairs for each of the 134 style clusters we identified in the final round data.

Finally, the ultimate Mask R-CNN segmentation model is trained on the combined augmented and rescaled training data. All images are adjusted such that the estimated nuclei size is uniform. To refine the segmentations for high pixel-level accuracy, the edges of each detected nucleus are corrected using a U-Net-based model trained on the same data, followed by some mathematical morphology-based post-processing (see STAR Methods). This step may be skipped if such accuracy is unnecessary for the application, for example, if simply counting nuclei.

## RESULTS

**Figure 2.**
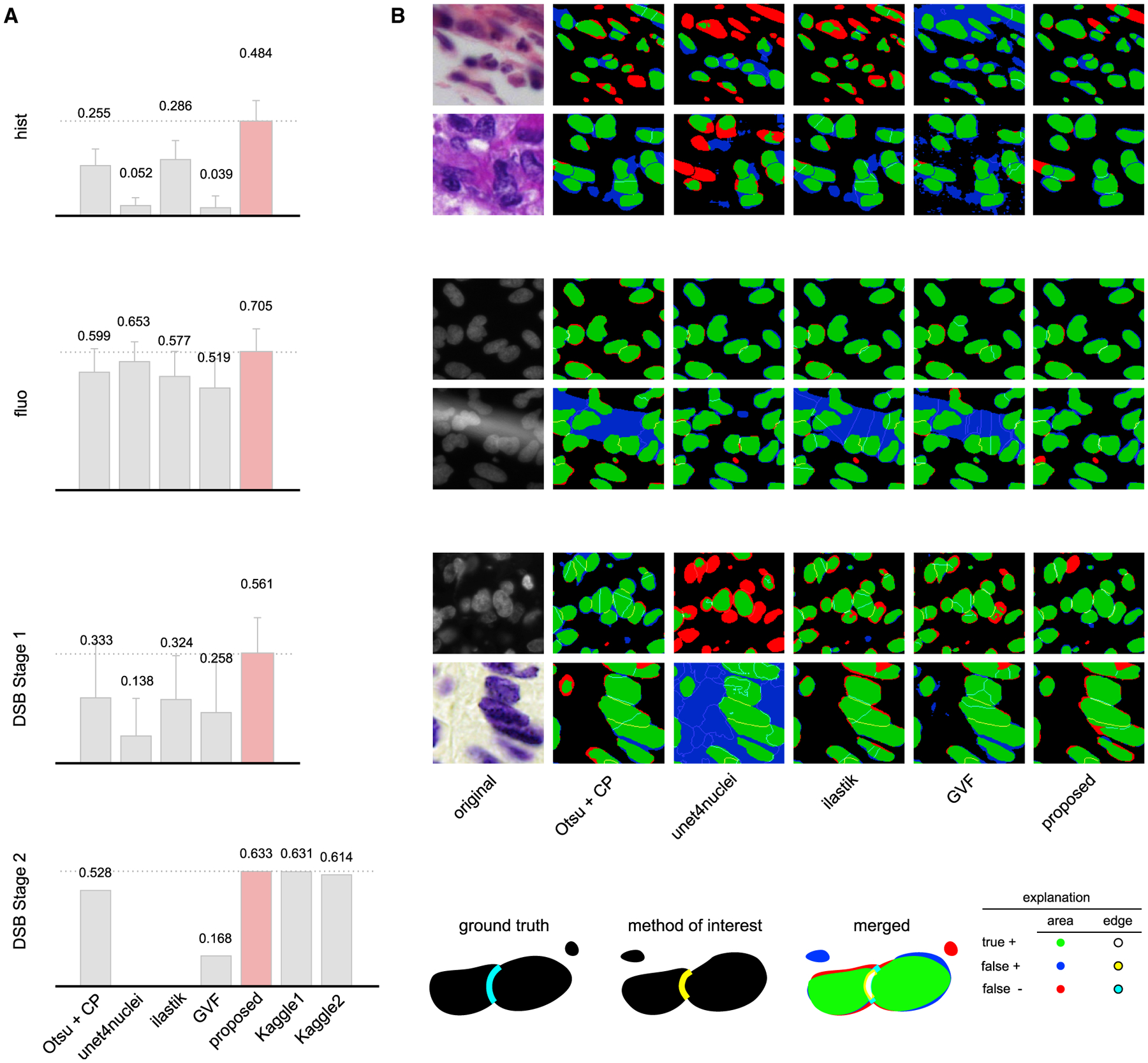
Results (A) DSB-scores with error bars (standard deviation) for four image sets: hist, fluo, DSB stage 1, and DSB stage 2 (see details in STAR Methods). DSB-score is a modified mean average precision of segmented nuclei (see STAR Methods). Highest scores are marked with dashed lines and red color. (B) Segmentation results for various methods on sample image crops with difficult cases (two example images of each); rows match those of (A) (note: ground truth is not public for DSB stage 2). A crop of the original image is provided in the first column, followed by segmentation results predicted by various methods. The color coding of the results is explained in the legend at the bottom. See also Figures S2, S5, and S8; Table S1.

We evaluated our approach on four different datasets: DSB stage 1, DSB stage 2, our own set of fluorescence microscopy images, and our own set of histology images from various sources (*DSB1, DSB2, fluo*, and *hist*, respectively, details in Table S2). We compare our approach against submissions from other teams on *DSB1* and *DSB2* (nearly 3,000 in stage 1 and 739 in stage 2). As benchmarks, we include the results reported in the first and second positions of the leaderboard, which was frozen at the close of the competition (https://www.kaggle.com/c/data-science-bowl-2018/leaderboard), a recent deep learning method, unet4nuclei (Caicedo et al., 2019a), which is based on a U-Net (Ronneberger et al., 2015) structure, a widely used Otsu threshold and seeded watershed method with object splitting (Carpenter et al., 2006), the pixel-based classification software ilastik (Sommer et al., 2011), and a more sophisticated but still classical gradient vector flow (GVF) based method, where an active contour is driven to edges using gradient vectors pointing to bright regions (Li et al., 2008) (Figure 2; Table S1; Data S2). Notably, the DSB stage 2 evaluation is performed on an unknown subset of the provided test images, many of which are outside the domain of the training images, truly challenging the ability of the model to generalize. We provide additional benchmarks and variations of our approach for comparison—including how our proposed style transfer learning step improves performance—in STAR Methods and Figure S2. Training a model on the same data with and without style transfer augmentation showed increased accuracy with style.

Our method scores higher (DSB-score, 0.633) than the top ranked deep learning approach (0.631, the highest of 739 teams) on the DSB stage 2 test set and has a simpler architecture with fewer parameters. Our method outperforms all other tested methods, too, including a classical baseline (0.528) (Caicedo et al., 2019b; Carpenter et al., 2006) (Figure 2A). In addition, our proposed method outperformed all prior published results on *hist*, a diverse set of histology images and on *fluo*, a fluorescent image set (BBBC039; Caicedo et al., 2019a) (see Data S1 and S2 for details). A detailed comparison of our results against six other methods evaluated with additional metrics is provided in Table S1; Figures S5 and S8 (see details in STAR Methods).

## DISCUSSION

We proposed a deep-learning-based nucleus segmentation approach designed for robustness to new experimental settings, using image style transfer to augment our training data with valuable out-of-domain samples. Our segmentation network learned from these artificially generated image/mask pairs, which mimic the patterns of new data types. This approach helped the network adapt to a diverse set of test data outside the domain of the training data, outperforming every other deep learning and classical method tested. Our generalized models successfully segment images across several domains, achieving performance close to or matching that achieved by models derived from and applied to a specific domain. The idea of augmenting difficult-to-obtain data using style transfer has enormous potential not only for nucleus detection but also more broadly in applications requiring some form of image understanding.

### Key Changes Prompted by Reviewer Comments

The manuscript was extended with the section Segmentation Error Analysis describing both advantages and limitations of our approach compared with other methods, while practical runtime and resource details were also given in section Methods Used for Comparison for training and inference so that the reader might have a better overview of applicability. Specific algorithmic considerations were clarified more extensively, e.g., clustering and image style transfer or post-processing. For context, the complete transparent peer review record is included within the Supplemental Information.

## STAR★METHODS

### RESOURCE AVAILABILITY

#### Lead Contact

Further information and requests for resources and reagents should be directed to and will be fulfilled by the Lead Contact, Peter Horvath (horvath.peter@brc.hu).

#### Materials Availability

This study did not generate new unique reagents.

#### Data and Code Availability

The authors declare that the data supporting the findings of this study are available within the paper and its Supplemental Information files.

The authors also declare that the software supporting the findings of this study are available within the paper, its Supplemental Information files, under www.nucleaizer.org, and https://github.com/spreka/biomagdsb.

### EXPERIMENTAL MODEL AND SUBJECT DETAILS

#### Kaggle Competition

We designed our pipeline to recognize nuclei as accurately as possible in a wide variety of images acquired with different microscopes, under varying imaging conditions with different stains for nuclei of various cell types. This was the challenge set forth in the 2018 Data Science Bowl (DSB) by Kaggle, Booz Allen Hamilton and the Broad Institute. The competition included a preparatory stage 1, to which teams could submit their solutions during a four-month period and a 4-day long stage 2 final scoring period.

Existing nucleus segmentation methods do not generalize well, they perform well only on the limited experimental conditions they are designed or tuned for. The Data Science Bowl was highly successful in the sense that many robust solutions were developed that pushed the state-of-the-art in terms of segmentation performance and insensitivity to image type and quality. Solutions such as ours are now being developed into toolkits for biologists that will accelerate science by improving automation in identifying nuclei.

We participated in the competition in both stages, reaching the top 1% in stage1 and top 4% in stage 2. The presented results are based on further improvements post-competition.

#### Data

The official dataset for the challenge is composed of a training set and two tests sets, one for each stage. The number of images in each set is 670 (training), 65 (stage 1 test), and 3019 (stage 2 test), stage 1 test masks were released in the second stage. The final evaluation of the teams’ performance was measured on a subset of the stage 2 test set (the identity of the subset remained hidden to the competitors). Many of the competitors used additional data besides the provided training data, as this was permitted as long as participants shared their sources on the official competition website (https://www.kaggle.com/c/data-science-bowl-2018). Our annotated training data included 12 additional data sources besides the DSB data, including some data sources annotated by experts in our institution. This extended the total number of training image/mask pairs from 735 to 1,102, and the number of annotated nuclei from 33,814 to 80,692 (not including the synthetic data). A summary of the data we used is provided in Table S2.

Using style transfer, we augmented our training data with synthetic image/mask pairs generated in the style of k=134 clusters of images from the DSB Stage 2 set, as described in Sections Clustering to Synthesizing new image/mask pairs. This added 2,680 synthetic image/mask pairs to the training data (approximately 263,701 annotated nuclei).

We tested various versions of our method along with several competing methods on four test datasets: *DSB test1*, *DSB test2*, *fluo*, and *hist*. DSB test1 and DSB test2 are heterogeneous test sets from the Kaggle challenge (stage 1 and stage 2). The *fluo* dataset is fluorescence images of U2OS cells in a chemical screen taken from the Broad Bioimage Benchmark Collection (BBBC039) (Caicedo et al., 2019a). The *hist* dataset is a mixture of histology images collected from the internet and prostate H&E stained slides collected in-house.

A fraction of the histological images manually annotated in our lab were used as test set *hist* (see Supplemental Data). BBBC039 (Caicedo et al., 2019a) images were used to train a fluorescent segmentation model, we refer as *fluo*. The *hist* and *fluo* test sets are disjoint from the respective training data.

We carefully prepared our test sets for evaluation by automatic clustering as follows. Each test set was split into disjoint parts; one was completely held out of all training procedures and solely used for evaluation, while the remaining part served as out-of-domain unannotated data, was clustered by *k*-means and forwarded to style transfer and subsequent training steps.

We collected histopathology images of test set *hist* intentionally from such experiments that lacked similar instances in our entire training set to test how well our approach would perform on various out-of-domain experiments. Hence, only style transfer learning could be used to input these missing domains’ information to our segmentation network.

All input images were initially converted to 8-bit 3-channel RGB images in.png format as well as images produced by our pipeline (except masks).

#### Computational environment

##### Software

Our pipeline is implemented using a shell script to allow continuous execution of the entire pipeline. Python 3 scripts execute the training and inference of Mask R-CNN, U-Net, and pix2pix which rely on the TensorFlow, Keras, and PyTorch environments. The clustering, post-processing, and initial steps of style transfer are implemented in Matlab. Our software is available for download at: https://github.com/spreka/biomagdsb where a detailed documentation can also be found discussing the required versions of frameworks and details about the architecture parameters.

The entire pipeline can be run both under Linux and Windows. In a typical use case, it is not necessary to retrain any of the models. Calling the *postComp* method without post processing provides excellent results. For specific experiments with no ground truth annotations, performing the style transfer learning part of our pipeline generates new synthetic training data in the missing domain on which training a new model results in fine segmentation. Alternatively, an online version of our method is available at www.nucleaizer.org.

##### Hardware

Our methods were trained and tested on a variety of Nvidia graphics cards, including GTX 1070, 1080Ti, and Titan Xp.

#### Related work

##### Mask R-CNN

He et al. (2017) published Mask R-CNN as an extension of Faster R-CNN to allow simultaneous instance detection and segmentation. The network architecture is similar to that of Faster R-CNN: feature extraction uses ResNet (50 or 101 layers) or alternatively Feature Pyramid Network (FPN), while head is as in Faster R-CNN extended with a fully convolutional mask prediction branch. A detailed discussion of extended R-CNN versions can be found in Weng, 2017.

We decided to incorporate Mask R-CNN in our pipeline due to its robustness, scalability and instance-awareness. It is currently one of the leading computational architectures in instance segmentation of arbitrary object classes, and its applications dominated the methods submitted to the DSB 2018 competition alongside solutions based on U-Net.

##### U-*Net*

U-Net (Ronneberger et al., 2015) was specifically created for bioimage segmentation with an encoder-decoder architecture and skip connections between layers of the encoding branch and decoding branch to provide the decoder with access to spatial information to reason about upsampling the segmentation.

We applied U-Net in our post-processing pipeline as it can efficiently be used to detect subtle differences such as those around the edges of objects. The network structure is straightforward and computationally feasible.

Post-processing the segmented nuclei per se is needed due to the inevitable uncertainty in marginal cases, like relatively small objects most likely corresponding to false detections. We found probability maps predicted by U-Net helpful in such scenarios.

### METHOD DETAILS

#### Overview of the Pipeline

As a first step, pre-segmentation of the input images is performed using a pre-trained deep convolutional model (which we refer as *preseg*) to estimate nuclei sizes as well as to create a mask input for image style transfer learning. Simultaneously, we cluster similar images of the input data into groups, and learn styles on these clusters (see Figure 1B and sections Clustering for Style Transfer Learning and Learning Image Style Transfer Models for details). As a next step, we extend the training data with artificially created style transferred images for fine-tuning a Mask R-CNN (He et al., 2017) pre-trained on our nucleus segmentation dataset. For inference on unseen data, we use the refined Mask R-CNN network incorporating knowledge about estimated cell sizes. The resulting contours are refined with U-Net (Ronneberger et al., 2015) and a morphology step.

The proposed method consists of procedures for training and inference, as shown in Figure S1. Inference merely requires unannotated images as its input – provided the pre-trained models are available. Training the network produces a learned segmentation model, and requires a set of annotated training data and a pre-trained segmentation network (pre-segmentation network), as well as any available unannotated images that can be used for data augmentation. The pretrained segmentation network is crucial to both the training and inference procedures, so we discuss it first and then continue with training and prediction steps.

#### Training and Style Transfer Data Augmentation

##### Pre-segmentation

The architecture for the segmentation networks is based on the Mask R-CNN architecture. The pretrained segmentation network (pre-segmentation network) is used to make rough estimates about the nucleus size and shape while being robust to changes in imaging modality or magnification. The network is initialized with pretrained weights from the MS-COCO dataset, which contains images and segmentation masks for 91 object types including people, trucks, sheep, dogs, etc. For details about the original COCO competition see http://cocodataset.org or the corresponding publication (Lin et al., 2014). The network was trained using a diverse set of annotated images containing various imaging modalities, cell lines, magnifications, etc. For more information see Section Data. The annotations consisted of segmentation masks for the nuclei. Augmentation was used during training including geometric transformations, intensity stretching, cropping, noise, and blur (see Data S1 documentation for details).

The resulting network, which we refer to as *preseg*, already performed reasonably well on unannotated images in the test set (Figure S2), although this was not its purpose. The preseg network is used to: estimate properties of nuclei in new unannotated images (size, shape, and area) in clustering, and to generate rough segmentations on unannotated images for the style transfer data augmentation step (see the following two sections for details).

#### Clustering for Style Transfer Learning

Images without annotations are automatically clustered to define multiple groups with similar properties: textures, imaging modalities, cell lines, sample type (tissue or culture), etc. These groups are used as data sources to learn style transfer models to generate additional synthetic data that mimics the properties of each cluster of unannotated images.

To perform the clustering, we use a pairwise similarity metric between feature vectors describing each unannotated image. Features were extracted using CellProfiler (Carpenter et al., 2006) modules including intensity and texture and a similarity metric was computed by a shallow fully connected neural network (Frank et al., 2016). This similarity network was trained on the DSB train1 data set, where images taken with the same condition are given a label of 1 and images from different conditions are given a label of 0. The output of this network on the unannotated data yielded a similarity matrix which we clustered with k-means. The number of clusters, k=134 for DSB stage 2 test set, was chosen automatically based on the number of images to over-segment the groups to avoid accidental mixing of the true underlying groups. Ideally, each obtained cluster of unannotated images represents a “style” or distribution of data which can be augmented with style transfer (e.g. digital slides of H&E stained breast cancer histology samples at 63x magnification, or fluorescent images of Human MCF7 cell nuclei at 40x).

##### Learning Image Style Transfer Models

We use the pix2pix (Isola et al., 2017) framework for image style transfer (https://github.com/junyanz/pytorch-CycleGAN-and-pix2pix). The architecture consists of two adversarial networks, a *generator* tasked with synthesizing realistic looking images, and a *discriminator* tasked with identifying real images from synthesized images. This model learns to map one image domain to another through an adversarial loss that encourages the generator to learn to fool the discriminator. The input to the generator in our case is a binary mask containing 1’s at the locations of the desired nuclei, and 0’s elsewhere. The input of the discriminator is an image/mask pair (either a real pair, or a synthetically generated pair). The generator learns to transform the binary mask into the desired style of the real images from the cluster, and the discriminator encourages this by trying to identify real image/mask pairs from fakes. We use the rough segmentations provided by the *preseg* network as masks for the unannotated images in the style cluster during learning. We train a pix2pix style transfer network to synthesize realistic images from masks for each of the style clusters.

##### Synthesizing New Image/Mask Pairs

Using our set of 134 trained style transfer networks, we synthesized 20 new image/mask pairs for each of the styles in the unannotated data. A crucial step for this task was to generate novel binary masks to provide as input to the style transfer network, which uses the mask to generate a realistic image of the cells with nuclei in the locations defined in the mask. We generated the masks algorithmically as a combination of 1) fetching real nuclei masks from a database, and 2) synthesizing nuclei using software (simcep; Lehmussola et al., 2007). Approximately 50% of the nucleus masks were created using each approach. In this manner, we generated 20 masks for each of the 134 style clusters, and then used the style transfer network to generate the corresponding images.

We assembled our nucleus mask database from images of the official DSB training set and further external datasets (see Table S2) - some of which we corrected for slight contour errors - and added each nucleus mask to the database. We fetched such nuclei masks that follow the features of the desired style and placed them on the synthetic mask images in accordance with the localization properties of the given style.

##### Training the Mask R-CNN Segmentation Network

The synthetic image/mask pairs generated by the style transfer network were added to the annotated training data to update the Mask R-CNN segmentation network. We used the implementation of Matterport (https://github.com/matterport/Mask_RCNN) and wrote handler scripts in Python to create the appropriate data structures and call functions. Training was performed in 3 steps with decreasing learning rate and targeted different layers of the Mask R-CNN network, as described in the documentation of the aforementioned Matterport repository.

The loss function was as defined in (He et al., 2017): it comprises of classification, localization and segmentation mask losses: L=L_cls_+L_box_+L_mask_ by ROIs, and defines mask loss as follows. Given the *k*-th region does belong to ground truth class *k* it takes the average binary cross-entropy loss which is formulated as

(Equation 1) $$L_{\text{mask}}\;=\;-\frac{1}{m^{2}}\sum_{1<i,j<m}\left[y_{ij}\;\cdot \;\text{log}{\hat{y}}_{ij}^{k}\;+\;\left(1\;-\;y_{ij}\right)\text{log}\left(1\;-\;{\hat{y}}_{ij}^{k}\right)\right]$$

where *y*_*ij*_ is the true label of a cell (*i,j*) from a ROI of *m*×*m* size on the ground truth mask of class *k* and ${\hat{y}}_{ij}^{k}$ is the predicted class label of the same cell. The formula only includes masks for ground truth class *k* that are associated with the *k*-th class.

##### Image Augmentation and Resizing

The performance of deep learning networks is known to scale with the size of the dataset (Hestness et al., 2017). Therefore, we use a number of approaches to augment the training data. The first, as we described above, is to add new synthetic image/mask pairs generated in the style of unseen examples to the existing annotated training data. Each minibatch contained 10–50% synthetic images. We also used standard data augmentation techniques including random cropping, colour channel swapping, intensity modification by histogram stretching or equalization and inversion, rotation to an arbitrary degree and translation as geometric transformations and finally, to better resemble low-quality images, blur and additive noise were used as well. These operations were applied to all the input training data – style transfer results too – with a random probability.

MASK R-CNN is reasonably robust to changes in scale, but superior performance is obtained if the nucleus size is approximately 40 pixels in diameter for the data and parameters we used. Figure S3 shows the results of the robustness of our method with a fixed parameter against different nuclei sizes. Quantitative evaluation is shown in Figure S6.

Another preprocessing step was to resize the images by a scaling factor to obtain a training dataset homogeneous both in cell and image size. The scaling factors were computed from the size estimation of the *preseg* nucleus masks such that the resulting mean cell size is set to 40 pixels diameter. Images were then either cropped or padded so that the resulting image was 512 × 512 pixels.

#### Inference

##### Mask R-CNN Prediction

The Mask R-CNN model trained as described above is used to predict segmentation masks when new images are provided as input. The images are resized before they are input to the network as described in the previous section.

##### Post-processing and U-Net Correction

We found that the segmentations could be further improved by postprocessing and refining nucleus contours using U-Net (Ronneberger et al., 2015). This encouraged better boundary reasoning between adjacent nuclei, and finer segmentations with the background. First, outlier objects were removed or merged as follows: 1) Smaller objects that were entirely within another object were eliminated. 2) objects that were surrounded by another object more than p_1_% were merged, and 3) objects smaller than p_2_ pixels area were removed. Next, U-Net based correction was performed (Figure S4): 1) an optimal threshold p_3_ for U-Net probability values was determined, 2) a soft margin around the Mask R-CNN contour was defined for each object, with an extension of p_4_ pixels inwards and p_5_ outwards. The contour was extended/shrunk based on the U-Net predictions. 3) objects that had in total less than p_6_ mean U-Net probability were removed. Parameters p_1_..p_6_ were optimized on the training set with a genetic algorithm to the DSB-score function (see formulation in section Evaluation Metrics). Best values were: (0.17, 44, 0.9375, 1, 1, 0.8).

### QUANTIFICATION AND STATISTICAL ANALYSIS

#### Evaluation Metrics

The evaluation metric used for the DSB competition is based on the mean average precision, as defined on the competition website, at different intersection-over-union (IoU) thresholds. A successful nucleus detection was determined by an IoU test (also known as the Jaccard index):

(Equation 2) $$IoU(x,\;y)\;=\;\frac{\left|x\cap y\right|}{\left|x\cup y\right|}\;=\;\frac{\left|x\cap y\right|}{\left|x|\;+\;|y|\;-\;|x\cap y\right|}$$

which measures the overlap between prediction pixels *x* and the annotation pixels *y* over the intersection of the two areas. Using a threshold ranging from 0.5 to 0.95 with steps of 0.05, true positive (*TP*) detections, false positive (*FP*) detections and false negative (*FN*) detections were identified. For a threshold of 0.5, a predicted object is considered a “hit” if the IoU is greater than 0.5. For each threshold *t*, a modified version of precision was calculated

(Equation 3) $$DSB\;\text{score}(t)\;=\;\frac{TP(t)}{TP(t)\;+\;FP(t)\;+\;FN(t)\;+\;\varepsilon }$$

for all thresholds in (0.5, 0.95). These scores were averaged for all thresholds, and then the mean of the average scores is reported over the images in the test dataset. In addition to the DSB-score, we evaluated our results with three additional metrics based on the IoU detection test: mean average precision- (mAP), recall and F1-score. We used the same *t*, TP, FP and FN values as above. We also added a small *ε* = 10^−40^ value to the denominators.

(Equation 4) $$\text{precision}(t)\;=\;\frac{TP(t)}{TP(t)\;+\;FP(t)\;+\;\varepsilon }$$

(Equation 5) $$\text{recall}(t)\;=\;\frac{TP(t)}{TP(t)\;+\;FN(t)\;+\;\varepsilon }$$

(Equation 6) $$F1\;\text{score}(t)\;=\;2\;\cdot \;\frac{\text{precision}(t)\cdot \text{recall}(t)}{\text{precision}(t)\;+\;\text{recall}(t)\;+\;\varepsilon }$$

The same strategy was used to calculate mean values for these measures as was for the DSB-score, taking the average over various thresholds *t*, and the mean among the test images. In the following sections, we refer to these measures as mAP (mean average precision), mAR (mean average recall), and mF1 (mean average F1-score).

We also introduce classification accuracy regarding our style-transfer generated image quality evaluation as follows:

(Equation 7) $$\text{accuracy}\;=\;\frac{\sum \text{correctly}\;\text{classified}\;\text{instances}}{\sum \text{instances}}$$

#### Methods Used for Comparison

Our tests included several variations of our method along with six competing methods and several variations of our approaching using different style augmentation: *NOstyle* did not contain style augmented images, *AUTOstyle* used nuclei masks generated by the preseg network, and *GTstyle* used hand annotated ground truth to generate nuclei masks. CellProfiler (*CP*) (Carpenter et al., 2006) is a widely-used bioimage analysis software incorporating several methods to segment, measure and analyze cellular compartments. We created multiple pipelines for the different image types of the test sets – except for our fluorescent set which comprised of a single experiment. *Preseg* refers to our general scale-independent pre-segmentation model while *postComp* is our final refined post-competition submission (an *AUTOstyle* model customized for DSB test2).

We compared against several other approaches including *ilastik* (Sommer et al., 2011), which provides a pixel classification setup where users can manually annotate regions of the input images to desired classes and obtain predictions as either probability maps or segmented images. Segmentations were obtained by applying a threshold to probabilities from *ilastik* (with additional object splitting). *Unet4nuclei* (Caicedo et al., 2019a) is an implementation of the popular U-Net deep learning approach to segmentation. *GVF* (Li et al., 2008), or gradient vector flow, is an active contour-based segmentation method suitable if objects are bright regions on a dark background. Pipelines of these compared methods are provided in Data S2. *DSB1* and *DSB2* are the first and second place entries on the final Kaggle leaderboard. The approach from *DSB1* (https://www.kaggle.com/c/data-science-bowl-2018/discussion/54741) uses a very deep U-Net architecture along with prediction of touching borders. *DSB2* also uses a U-Net approach, and forces the network to predict relative locations within each nucleus (https://github.com/jacobkie/2018DSB).

Comparing the complexity as well as the computation time and resources needed to train *DSB1*, we are confident to claim that our method is considerably simpler and much faster. *DSB1* combines a total of 32 trained deep neural networks to achieve their reported score on *DSB test2* set, the training of which can take days even when performed on a high computation-capable GPU (Nvidia GTX 1080Ti). In contrast, in our method only a Mask R-CNN and U-Net models are trained for prediction, taking approximately 10 hours for training on the same GPU. The computation time for image style transfer strictly depends on the number of different styles present in the target data as one style model is trained for each, individually taking about 15 minutes. *DSB2* uses a simpler architecture.

We also investigated computation time regarding inference with our method. Even though inference time is affected by multiple circumstances including image size, number of objects on the image and VRAM of the GPU used, an approximate one image per 2 seconds can be achieved given the following. An image of 520×696 pixels size having about 120 objects of ~20 pixels median diameter size, rescaled to 2x its original size to have ~40 pixels diameter sized objects, i.e. 1040×1396 pixels resized image, on an Nvidia GTX 1080Ti GPU having 11 GB VRAM can be predicted in 2 seconds.

#### Detailed Results

##### Style Transfer Increases Performance

We tested the methods outlined in Section Methods Used for Comparison on four test datasets: *DSB test1*, *DSB test2*, *fluo*, and *hist*, described in Section Data. The resulting DSB-scores are presented in Table S1. When running these tests, the test data was never included in the data to train the model, e.g. when testing on *DSB test1*, the *DSB test1* data was held out from the training set. Similarly, when testing on hist, *biomag2* and *biomag6* subsets were held out.

The test image sets were used as style transfer learning input as determined by our automatic clustering method: a portion of the set was left out when the clustering algorithm could not find a sufficient number of images for a cluster. Therefore, we report our results on such fractions of the test sets that none of the deep learning networks have seen prior to inference as follows. 100/200 *fluo*, 21/50 *hist*, 28/65 *DSB test1* images were used for evaluation. None of the final *DSB test2* evaluation image set was used for training.

The results demonstrate that our style transfer approach improves performance in test sets containing data from heterogeneous sources: *hist*, *DSB test1* and *DSB test2*. We also see excellent performance on single domain fluorescence data, *fluo*. Comparing the results of our method with (*AUTOstyle* [postComp is the *AUTOstyle* for DSB stage 2 test] and *GTstyle*) and without style transfer augmentation (*NOstyle*), we see a clear trend towards increased performance with style transfer augmentation. If we have access to ground truth nucleus masks (*GTstyle*) our performance improves, though in many realistic scenarios such masks will not be available. Figure S2 shows the output of the various methods we tested on challenging examples (note that *DSB1* and *DSB2* are not reported because we did not have access to their code). In Figure S5, we present mAP, mAR, mF1 and mIoU metrics for the various methods on each dataset. As expected, there is a strong correlation between the metrics.

##### Objects of Various Sizes can Be Detected Accurately

In addition to the qualitative demonstration on Figure S3, we provide a quantitative analysis of the range of object sizes correctly detected by two of our compared methods: *preseg* and *postComp*. Note that while *postComp* was trained on fixed sized (40 pixels diameter) nuclei images and is expected to perform best on objects of approximately the fixed size, *preseg* is more flexible as we intentionally included images presenting a wide range of object sizes in its training to prepare it for an initial robustness. Therefore we expect *preseg* to detect objects robustly in a wider size range. We tested both models on DSB stage 2 test set and scaled the images to 0.25–4.0 times relative to our generally expected median 40 pixels diameter objects. Our results confirm our expectations of *preseg* (our scale-independent model) which performs significantly better than *postComp* (scaled model) on shrunk images as presented on Figure S6 below. We found that the accuracy of both models is decreased far less rapidly when enlarging the images.

We also note that the object sizes can vary on individual images (Figure S6B) suggesting the scaling procedure by median object sizes cannot necessarily be optimal for all images; we mark some of the extremes with black arrows.

##### Synthetic Images Are often Mistaken for Real

We tested how well our style transfer-generated synthetic images compared to real microscopy images by showing a representative selection of both to field experts (pathologists and biologists) and asked them to tell the synthetic images apart from the real ones. The only prior information forwarded to the participating experts was there are fake images in the collection. Their decision accuracy was measured in a binary fashion: whether the expert could identify a truly synthetic image (1) or not (0). We show an example test image montage below (Figure S7) with the average detection of experts and the labels (real or fake). We collected 64 cropped images each for our two test image mosaics comprising of 50% real and fake tiles, respectively.

We report an approximate 57% accuracy (ranging from 42% to 73%) of fake image recognition averaging both our experts and the test cases. Based on the performance of the experts we can conclude the visual quality of the style transfer-generated images is on par with real microscopy images suggesting the advance our approach may bring to cellular compartment segmentation.

##### Segmentation Error Analysis

We visually compare segmentation errors and improvements on Figures 2 and S2. To better understand the distribution of such common errors in any of the analyzed segmentation methods we compared how well they perform in terms of avoiding the main error types: 1) missing a nucleus, 2) falsely detecting an object as nucleus, 3) splitting a nucleus and 4) merging adjacent nuclei unnecessarily. An example image presented on Figure S8A shows them visually. All existing methods fail to overcome these issues in at least some instances, as they significantly depend on the experimental and imaging conditions used to produce the images. Our method aims to help reduce these issues.

We measured such types of errors as follows. 1) a missed nucleus is a false negative (FN) i.e. present on ground truth (GT) with no corresponding object on the prediction. 2) A falsely detected nucleus is a false positive (FP): a predicted object with no corresponding GT. 3) A split nucleus is identified as two or more predicted objects that overlap with a significant region of the best corresponding GT object, respectively; we considered an overlap of at least 30% as significant in this case if two objects contributed to the overlap, and 15% if more. Splits were only considered if the given GT object did not have a single matching predicted object. 4) A merged nucleus is a single predicted object that has a significant overlap with multiple GT objects each. We calculated merges similarly to splits but swapped the role of GT and predicted objects.

We conducted our evaluation on the same subsets of each test set discussed in the previous sections. Quantitative analysis of segmentation errors support our results: our method (and its modified versions) generally outperform the compared methods. Comparative results are displayed on Figures S8C–S8E. Remarkably, *unet4nuclei* produced in total fewer errors than our methods on test set *fluo* but it has been trained and published on this image set.

Segmentation errors naturally occur in automatic methods. Classical methods (*CP*, *ilastik*, *GVF*) tend to predict a higher frequency of false positive objects, typically on complex background regions similar to e.g. Figure S4C. They are also more prone to merging touching nuclei or background regions around them to the objects (see Figure S2B rows 1–2) and to split larger, irregularly shaped objects. *Unet4nuclei* could not have been trained accurately enough for heterogeneous sets (*hist*, *DSB test1*) due to the inevitable uncertainty of U-Net in complex histological regions while it excelled on the single-domain set *fluo*.

Our method typically failed to split (i.e. merged) very small or elongated adjacent nuclei with weak textural difference from the dividing background region. Similarly, it unnecessarily split nuclei in cases where texture or edge information may suggest multiple nuclei-like structures inside a single nucleus.

## Supplementary Material

Supplementary Table 1

Supplementary Table 2

Supplementary Figure 8

Supplementary Figure 5

Supplementary Figure 6

Supplementary Figure 7

Supplementary Figure 4

Supplementary Figure 1

Supplementary Figure 2

Supplementary Figure 3

Data S2

Data S1

## Figures and Tables

**Table:** KEY RESOURCES TABLE

REAGENT or RESOURCE	SOURCE	IDENTIFIER
Software and Algorithms		
Code repository	This manuscript	https://github.com/spreka/biomagdsb
NucleAIzer online tool	This manuscript	www.nucleaizer.org
CellProfiler plugin	This manuscript	https://github.com/CellProfiler/CellProfiler-plugins/blob/master/nucleaizer.py

## Highlights

- Robust method automatically adapting to various unseen experimental scenarios
- Deep learning solution for accurate nucleus segmentation without user interaction
- Accelerates, improves quality, and reduces complexity of bioimage analysis tasks
- Easy-to-use online tool provided for the method
