# Supplementary figures and images for: nucleAIzer: A Parameter-free Deep Learning Framework for Nucleus Segmentation Using Image Style Transfer
Source: Cell Syst. Author manuscript; Available in PMC 2021 Jul 1. (PMC8247631; doi:10.1016/j.cels.2020.04.003)

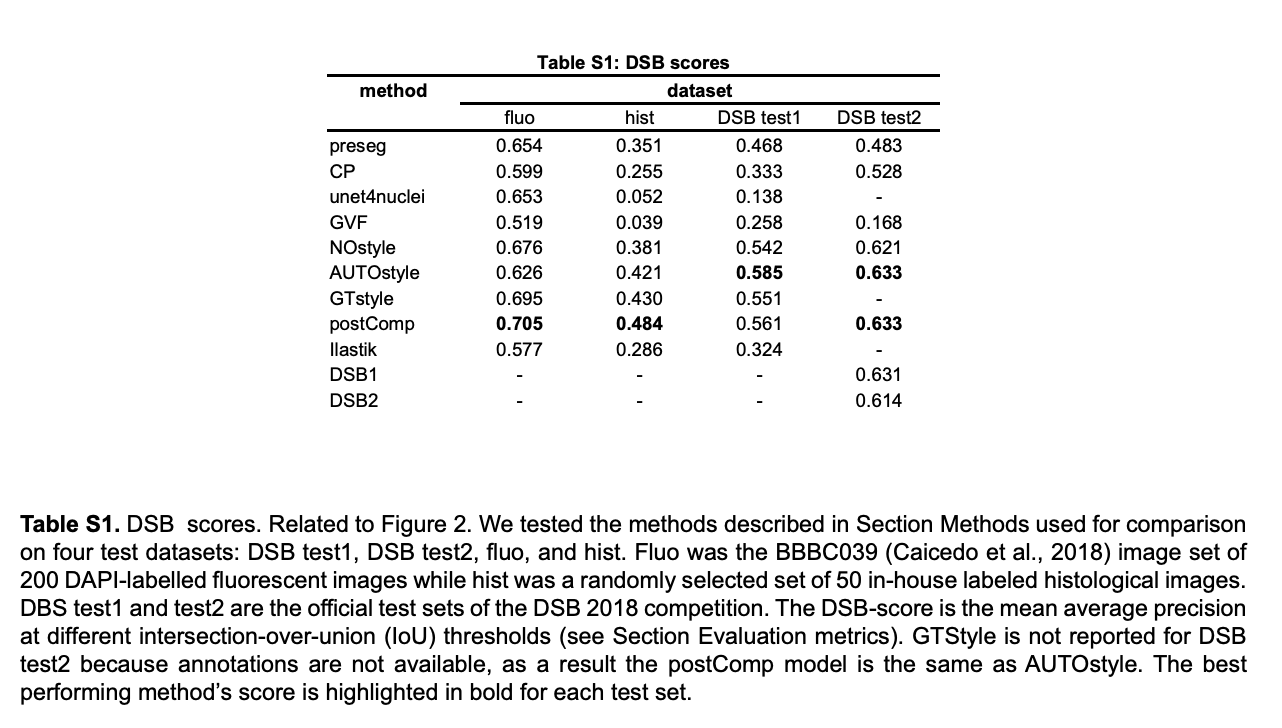

Supplement: Supplementary Table 1 [file NIHMS1679566-supplement-Supplementary_Table_1.png]

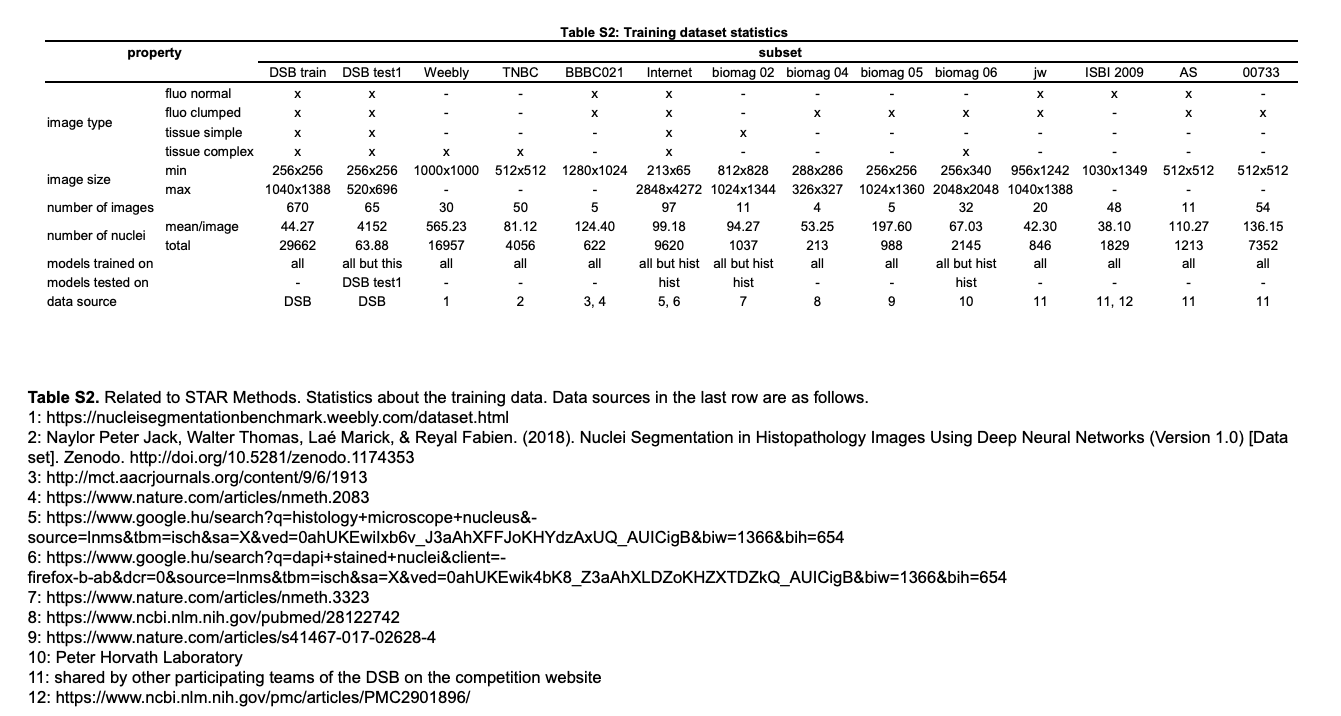

Supplement: Supplementary Table 2 [file NIHMS1679566-supplement-Supplementary_Table_2.png]

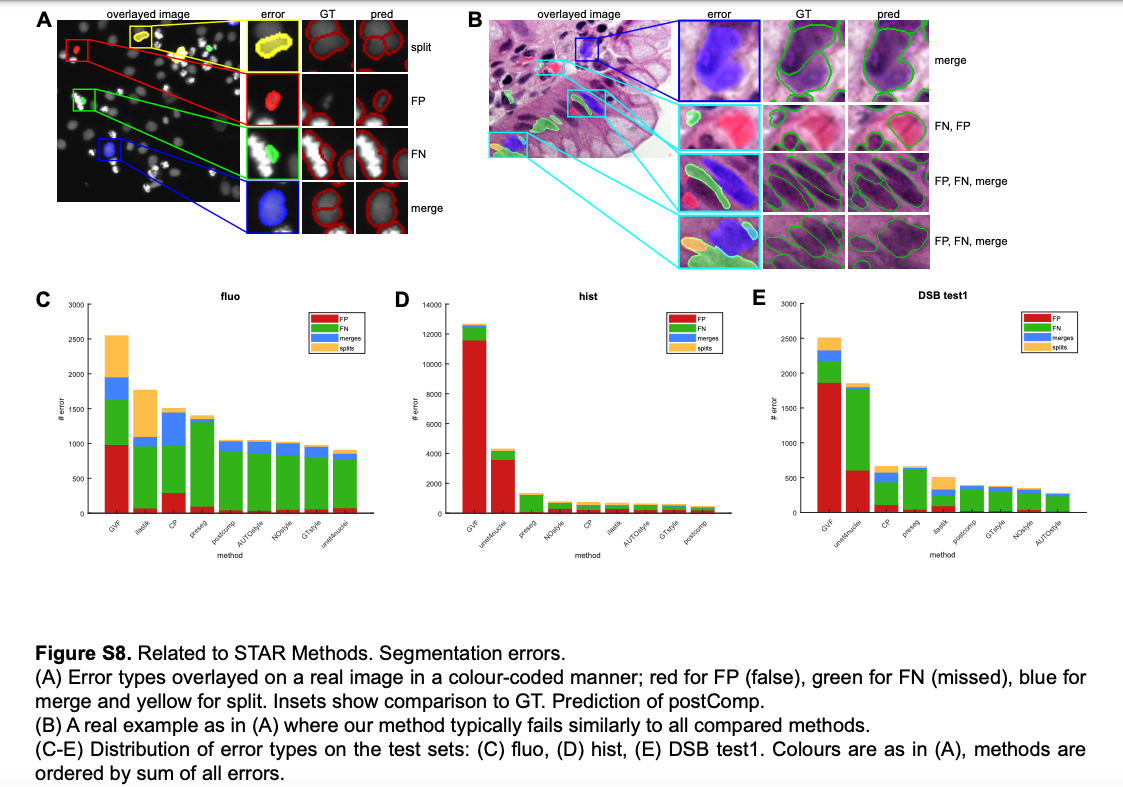

Supplement: Supplementary Figure 8 [file NIHMS1679566-supplement-Supplementary_Figure_8.png]

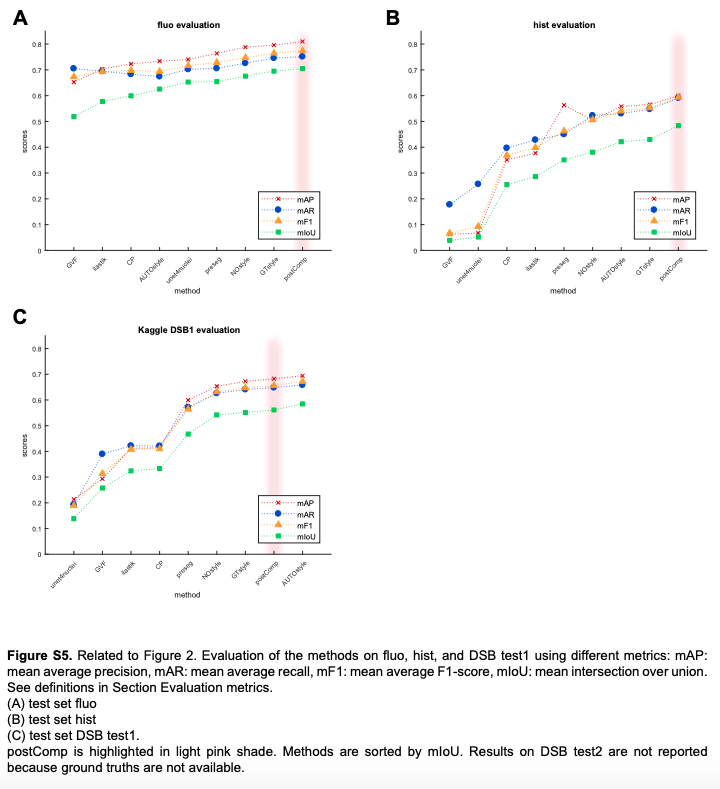

Supplement: Supplementary Figure 5 [file NIHMS1679566-supplement-Supplementary_Figure_5.png]

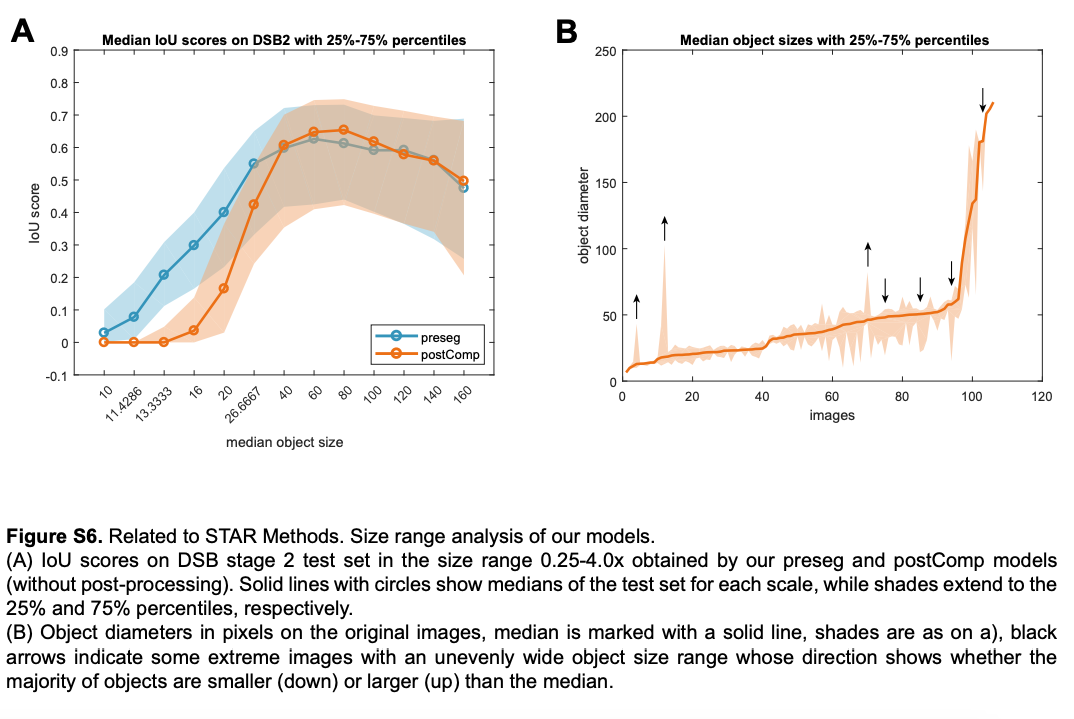

Supplement: Supplementary Figure 6 [file NIHMS1679566-supplement-Supplementary_Figure_6.png]

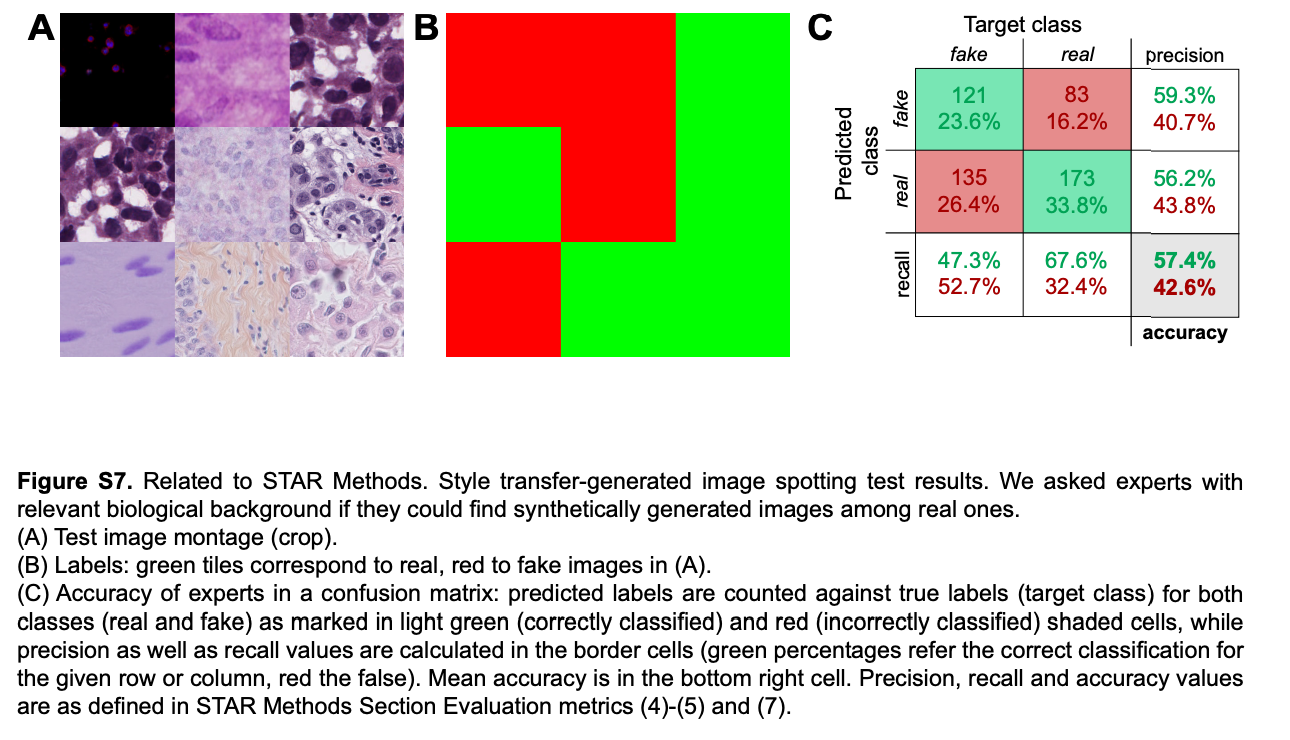

Supplement: Supplementary Figure 7 [file NIHMS1679566-supplement-Supplementary_Figure_7.png]

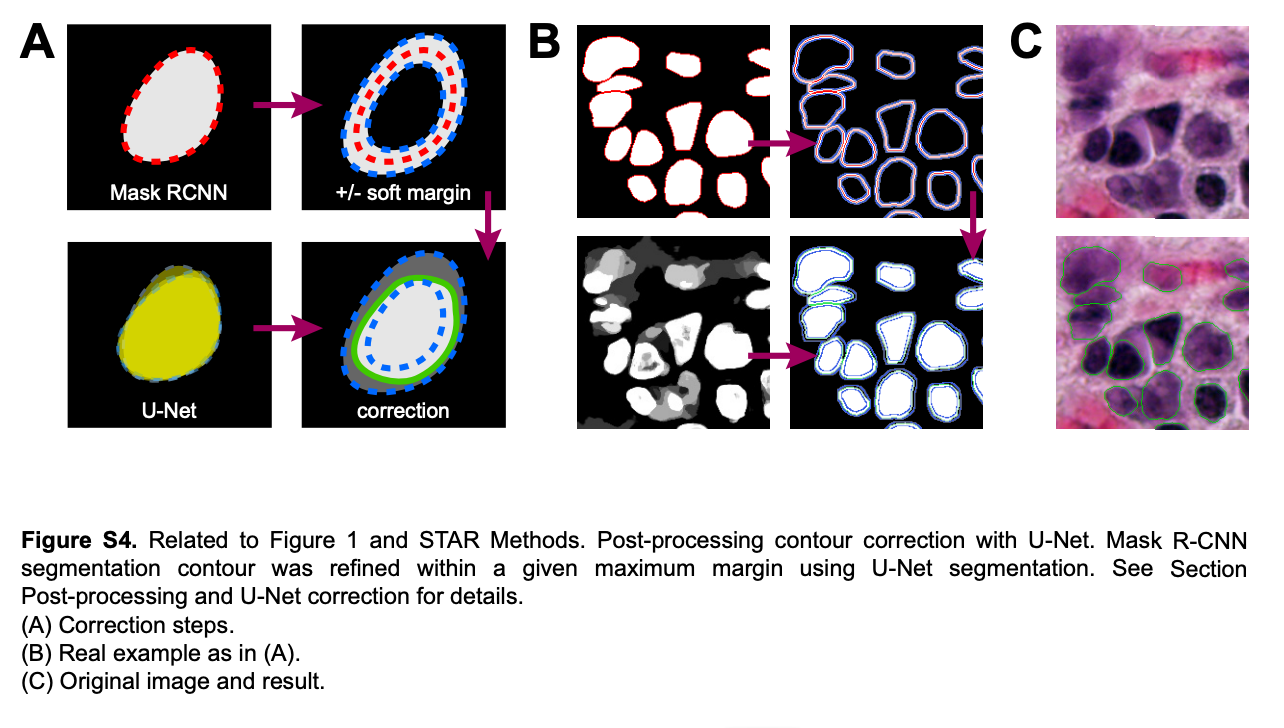

Supplement: Supplementary Figure 4 [file NIHMS1679566-supplement-Supplementary_Figure_4.png]

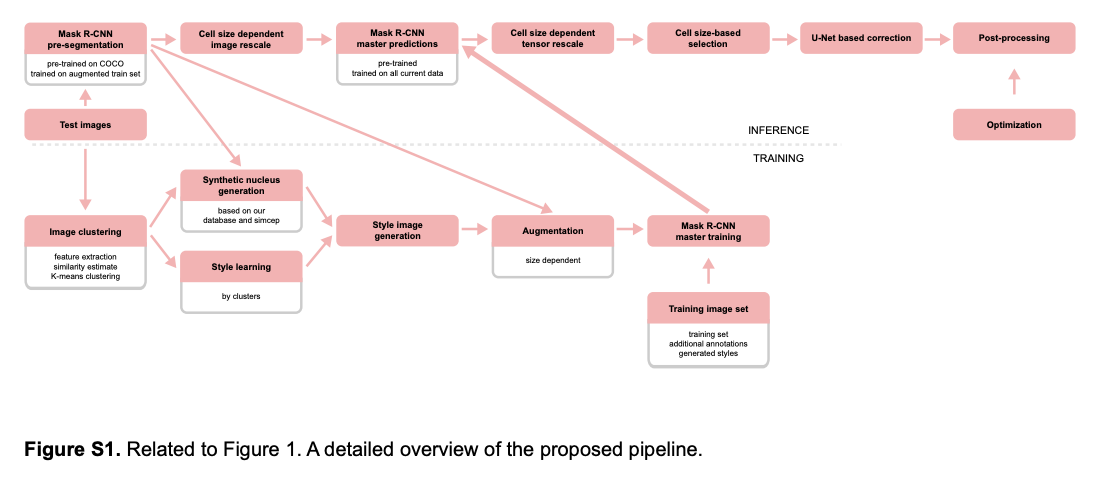

Supplement: Supplementary Figure 1 [file NIHMS1679566-supplement-Supplementary_Figure_1.png]

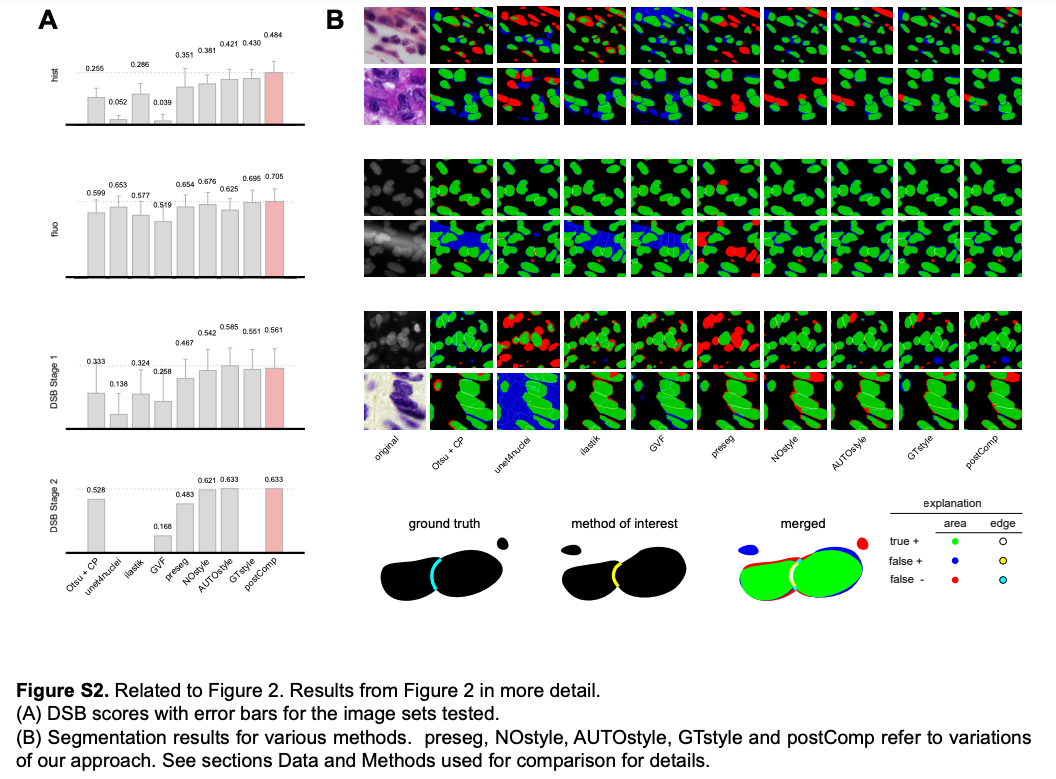

Supplement: Supplementary Figure 2 [file NIHMS1679566-supplement-Supplementary_Figure_2.png]

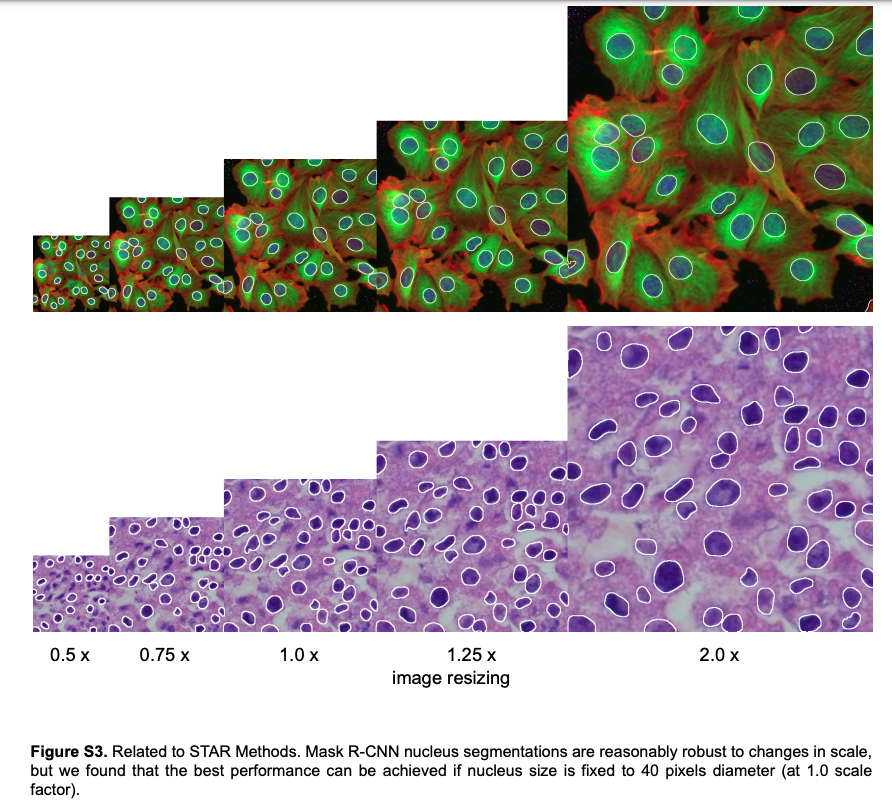

Supplement: Supplementary Figure 3 [file NIHMS1679566-supplement-Supplementary_Figure_3.png]
