# Supplementary material for: nucleAIzer: A Parameter-free Deep Learning Framework for Nucleus Segmentation Using Image Style Transfer
Source: Cell Syst. Author manuscript; Available in PMC 2021 Jul 1. (PMC8247631; doi:10.1016/j.cels.2020.04.003)
Supplement: Data S2 [file NIHMS1679566-supplement-Data_S2.zip › supplementary_software/pipelines/suppSoftware3_otherMethods_with_doc.zip!/Documentation.pdf]

Documentation for supplementary software of the paper “**A general deep learning framework for cell nuclei detection using image style transfer**”

Citation

Please cite our paper if you use our method:

[...]

Prerequisites

The supplementary software (except for GVF) are third-party software as follows:

CPOtsu - CellProfiler 3.0 pipelines; software: http://cellprofiler.org/ pipelines: [...]

ilastik - ilastik projects followed up by simple CellProfiler thresholding and object splitting; software: http://ilastik.org/ projects: [...]

unet4nuclei - a U-Net-based deep learning method; software: https://github.com/carpenterlab/unet4nuclei

GVF - gradient vector flow algorithm implemented in C++ followed up by simple Otsu thresholding in Matlab; codes: [...]

Note: code was tested on Windows 10 with Matlab 2017a.

Usage

CellProfiler pipelines

You need to install CellProfiler first, please follow instructions at the link above.

1. Open a new project
2. Load one of our CellProfiler pipeline files (.cppipe) or CellProfiler project file (.cpproj)
3. Load desired images
4. Run pipeline

ilastik pipelines

You need to install ilastik first, please follow instructions at the link above.

1. Open one of our ilastik project files (.ilp)
2. Add desired images
3. Export predictions as probability maps to desired location
4. Start CellProfiler
5. Load one of our corresponding CellProfiler pipeline files (.cppipe) or CellProfiler project file (.cpproj)
6. Load the probability images obtained in step 3.
7. Run pipeline

unet4nuclei method

You need to clone the git repository at https://github.com/carpenterlab/unet4nuclei and install the required python packages listed in \unet4nuclei\unet4nuclei_req.txt.

To train and predict on an image set, please follow instructions in the provided ipython notebooks of the repository.

GVF method

The GVF code was compiled on Windows, and for the final processing step also requires Matlab runtime. Please see https://www.mathworks.com/products/compiler/matlab-runtime.html and select the one appropriate for your system.

NOTE: the Matlab code for GVF was tested on Matlab R2017a. The GVF code requires grayscale .bmp files as input images.

The GVF code may be called from a matlab command provided the necessary input arguments are parsed correctly.

1. Call the function run_gvf_cpp_fcn_FINAL.m with the indicated inputs
2. To process the results, call the function postproc_gvf_beforeOtsu_FINAL.m
